# Relating outdoor play to sedentary behavior and physical activity in youth - results from a cohort study

**DOI:** 10.1186/s12889-021-11754-0

**Published:** 2021-09-21

**Authors:** Carina Nigg, Claudia Niessner, Claudio R. Nigg, Doris Oriwol, Steffen C. E. Schmidt, Alexander Woll

**Affiliations:** 1 Institute of Sports and Sports Science, Karlsruhe Institute of Technology, Engler-Bunte-Ring 15, 76131 Karlsruhe, Germany; 2 Institute of Sport Science, University of Bern, Bremgartenstrasse 145, 3012 Bern, Switzerland

**Keywords:** Sedentary behavior, Screen time, Physical activity, Outdoor play, Cohort study, Children, Adolescents

## Abstract

**Background:**

Outdoor play, sedentary behavior (SB), and moderate-to-vigorous physical activity (MVPA) are related to youth’s health, however, there are research gaps regarding 1) associations between outdoor play, SB, and MVPA across a broad pediatric age range (6–17 years), and 2) longitudinal associations between outdoor play, SB, and MVPA across childhood and adolescence. Two studies were conducted to address those research gaps: Study 1 aimed to investigate relationships between outdoor play and accelerometer-assessed SB and MVPA in a cross-sectional nationwide sample of children and adolescents in Germany. Study 2 aimed to investigate prospective associations between outdoor play and self-reported screen-time SB and MVPA and in a sample of children with three measurement timepoints across 11 years.

**Methods:**

Data were obtained of the German national representative Motorik-Modul (MoMo) Study and the German Health Interview and Examination Survey for Children and Adolescents (KiGGS). In Study 1, *N* = 2278 participants (6–17 years) were included with self-reported outdoor play and accelerometer-assessed SB and MVPA. Associations were examined via multiple linear regressions. In Study 2, *N* = 570 participants (baseline: 4–7 years) were included in the longitudinal analysis with follow-ups six and 11 years later. Screen-time SB (TV watching and PC/Gaming), MVPA, and outdoor play were self-reported. Associations were investigated through a path prediction model.

**Results:**

Study 1 showed that compared to <1 h outdoor play, higher engagement in daily outdoor play was related to lower SB (1-2 h: − 9.75 min/day, *P* = 0.017; ≥2 h: − 17.78 min/day, *P* < 0.001) and higher MVPA (≥2 h: + 3.87 min/day, *P* = 0.001). The cross-sectional relationship between MVPA and outdoor play was moderated by sex (in favor of males) and age (in favor of younger children). Study 2 showed that outdoor play in early childhood negatively predicted PC use/Gaming in later childhood, but was unrelated to MVPA.

**Conclusion:**

In Study 1, outdoor play was negatively related to SB cross-sectionally. In Study 2, outdoor play in early childhood was negatively related to PC and Gaming time in later childhood. Thus, providing outdoor play opportunities, especially during early childhood, has potential to prevent SB. Future research should investigate longitudinal relationships using device-based assessments for SB and MVPA.

**Supplementary Information:**

The online version contains supplementary material available at 10.1186/s12889-021-11754-0.

## Background

Outdoor play, sedentary behavior, and moderate-to-vigorous physical activity are related to youth’s health [1–5]. Sedentary behavior describes any behavior in a sitting or declined position requiring less than 1.5 metabolic equivalents [6], and physical activity describes any skeletal muscle movement leading to energy expenditure [7]. While increased sedentary behavior is related to physical, behavioral, and psychosocial health problems [1, 5], increased physical activity is associated with numerous physical and mental health benefits in youth [2]. Thus, the World Health Organization recommends limiting sedentary behavior, specifically screen-time sedentary behavior, in children and adolescents, and recommends that children and adolescents engage on average in at least 60 min moderate-to-vigorous physical activity a day across the week [8]. However, prevalence data show that youth across the US and Europe spend between 4 and 12 h a day sedentary [9] and that globally only about 20% of youth conduct at least 60 min moderate-to-vigorous physical activity daily [10], which is similar to physical activity levels in Germany when assessed via self-report [11].

An activity especially relevant for children is outdoor play, defined as any unstructured physical activity taking place outdoors during children’s leisure time [12]. In children between two and five years, outdoor play was positively related to prosocial outcomes, such as openness and cooperation [3, 4]. In three- to four-year-old preschoolers, more outdoor play was related to a body-mass-index (BMI) decrease and a reduced risk for obesity at the end of the preschool year [13]. In another study with about 20,000 Canadian youth in grades six to 10, Janssen [14] showed that replacing one hour active outdoor play with one hour active video gaming per day was associated with a 6% reduced probability of prosocial behaviour, a 3% reduced probability of high life satisfaction, and a 7% increased probability in high emotional problems. In the Canadian Health Behaviour of School Children study with youth aged 11–15 years, more than 30 min outdoor play were related to a 24% decrease in the prevalence of psychosomatic symptoms, including feeling low and nervous, sleep problems, and bad temper in females, while no significant associations were observed for males [15]. Although outdoor play is related to several health benefits, across the last two decades, older children and adolescents in Germany decreased outdoor play: Between 2003 and 2006, 62% of children (11–13 years) and 37.2% of adolescents (14–17 years) engaged in more than three days outdoor play per week, however, between 2014 and 2017, this decreased to 50.1% and 14.6%, respectively, with similar trends for boys and girls [16], whereas outdoor play remained stable in children between four and ten years with 79.8%–91.2% engaging in outdoor play on more than three days per week [16]. Thus, although the highest outdoor play levels are observed in children up to 10 years, the data shows that outdoor play is still salient in older children and adolescents [16].

As outdoor play is understood as a behavior that includes activity [12], several studies investigated relationships between acute sedentary behavior, physical activity, and outdoor play. A meta-analysis of mostly cross-sectional studies revealed that children (two to five years) in childcare centers spent 44% of outdoor play time in total physical activity, but only 14% in moderate-to-vigorous physical activity, whereas 53% of outdoor play time was sedentary based on accelerometer measurement [17]. Other studies investigated associations between habitual physical activity, sedentary behavior, and outdoor play. A recent systematic review about correlates of outdoor play in children between three and 12 years supported positive associations between outdoor play and physical activity, while there was largely no relationship observed between screen time or screen exposure as a proxy for sedentary behavior and outdoor play [18]. Again, most of the studies were cross-sectional and most of the studies looked at children in preschool age [18]. Fewer studies investigated associations between habitual outdoor play, moderate-to-vigorous physical activity, and sedentary behavior in older children: In 11-year-old children, high levels of outdoor play were associated with more moderate-to-vigorous physical activity and less sedentary behavior on weekend days for boys and girls, while on weekdays, these associations were only observed for boys [19]. In another cross-sectional study with six-to-13 year-olds, engaging in outdoor play on one or more days per week was associated with a 23–62 min increase in moderate-to-vigorous physical activity in boys, while those associations were not observed for girls [20]. A long-term follow-up study investigated participation in outdoor play in ten-year-old children and sports and physical activity participation at the age of 42 years, showing that outdoor play was not predictive of physical activity in adulthood [21]. However, this study did not investigate longitudinal associations across the developmentally important period of childhood and adolescence.

In summary, several studies have shown a positive relationship between acute and habitual outdoor play and physical activity cross-sectionally, with most investigations targeting children in the elementary school age, while there is little known about the relationship between outdoor play and sedentary behavior. Thus, there are research gaps regarding 1) associations between outdoor play, sedentary behavior, and physical activity, and in a broad age range of children and adolescents, and 2) longitudinal associations between outdoor play, sedentary behavior, and physical activity across childhood and adolescence.

The aim is to address those research gaps with two studies. To address the first research gap, Study 1 aims to investigate relationships between outdoor play and accelerometer-assessed moderate-to-vigorous physical activity and sedentary behavior in a cross-sectional sample of children and adolescents between six and 17 years. Study 2 aims to address the second research gap by investigating prospective associations with outdoor play and self-reported moderate-to-vigorous physical activity and screen-time sedentary behavior in a sample of children with three measurement timepoints across 11 years. The different measurement instruments are due to the fact that accelerometer data were not available at the first two measurement time points.

The STROBE statement [22] guided the reporting of this study.

## Methods

### Study 1

#### Procedures

Data were obtained from the Motorik-Modul (MoMo) study, an in-depth cohort study [23] within the German Health Interview and Examination Survey for Children and Adolescent (KiGGS) conducted by the Robert Koch Institute [24]. For the current study, three measurement timepoints were used: Baseline data (T1) were collected from 2003 to 2006, the first follow-up was from 2009 to 2012 (T2), and the second follow-up from 2015 to 2017 (T3) [23]. At all measurement timepoints, data was collected across the whole year. Study participants were selected based on a multi-stage sampling approach with two evaluation levels [25]: First, a systematic sample of 167 primary sampling units was selected from an inventory of German communities and stratified according to the level of urbanization and geographic distribution. Second, based on the official registers of local residents, an age-stratified sample of randomly selected children and adolescents was drawn. Participation in the study was voluntary. Participants and their parents were informed about the study’s aims, contents, and data protection, and gave written consent. At all measurement time points, parents and children were invited to examination rooms within proximity to their homes for data collection. For children younger than 11 years, parents completed the questionnaires.

The study was conducted according to the Declaration of Helsinki. Ethics approval was obtained by the Charité Universitätsmedizin Berlin (T1), the University of Konstanz (T2), and the Karlsruhe Institute of Technology (T3).

#### Participants

For Study 1, only data from MoMo T3 were utilized as this was the only occasion where accelerometer-measured sedentary behavior and moderate-to-vigorous physical activity were available. To be eligible for Study 1, participants had to be part of MoMo T3, younger than 18 years, and participate in the accelerometer measurement. In MoMo T3, 4569 youth between six and 17 years participated. A total of 2734 participants agreed to wear an accelerometer. Of those, 465 datasets were not considered as valid (see measures section), resulting in 2278 study completers (52.9% female, mean age 12.52 years [SD = 3.30], 73.7% normal weight and 17% overweight or obese, 64.5% middle and 28.0% high socio-economic status; see Table 1). Study completers were slightly younger and more likely to be female and have a normal weight. Detailed information about the differences between study completers and non-completers can be found in the *Additional File (A1)*.

**Table 1:** Socio-demographic characteristics and weight status of participants in Study 1 (*N* = 2278)

	%	N	% missing
*Sex*			0.0
Female	52.9	1206	
Male	47.1	1072	
*BMI*			3.4
Underweight	9.3	204	
Normal weight	73.7	1622	
Overweight	13.2	290	
Obese	3.8	84	
*Socio-economic status*			0.4
Low	7.5	170	
Middle	64.5	1462	
High	28.0	636	

Abbreviation: BMI = body-mass-index

#### Measures

##### Socio-demographic variables and weight status

Variables assessed included age, sex (male/female), socio-economic status, and weight status. The socio-economic status is a multidimensional score that is created based on information of both parents regarding education, occupational status, and net income. For children with separated parents, the socio-economic status of the parent they live with was used [26]. Participants who are in the first quintile of the score are categorized as participants with low socio-economic status, participants in the second to the fourth quintile are categorized as middle socio-economic status, and participants in the fifth quintile as high socio-economic status [26]. Height and weight were assessed by trained staff and BMI categories were established based on the cut-off points of the International Obesity Task Force [27].

##### Physical activity and sedentary behavior

Accelerometer use in this study is described somewhere else in detail [28]. Cross-sectional participants aged six to 17 years of MoMo T3 were asked to wear an accelerometer (ActiGraph GT3x + or ActiGraph wGT3X-BT) for seven consecutive days. Participants were instructed to place the accelerometer laterally on top of the right anterior superior iliac spine and to wear it during waking hours. Data was sampled using a frequency of 30 Hz. Downloaded data were converted into one-second-epochs and re-integrated into 15-s-epochs. Based on the Choi algorithm [29], non-wear time was defined as 90 min without consecutive zero/non-zero counts. To detect artificial movements, two-minute intervals of non-zero counts with the up−/downstream 30-min consecutive zero count windows were allowed [29]. To be considered as a valid dataset, participants had to wear the device more than eight hours on at least four weekdays and one weekend day. To determine moderate-to-vigorous physical activity and sedentary behavior, two cut-off point systems were applied that are commonly used for the specific age groups six to nine years [30] and for 10 to 17 years [31].

##### Outdoor play

Participants reported how many minutes a day they spent playing outdoors, such as playing tag or rope skipping. Minutes were then recoded into hours per day and based on the results of a frequency analysis and a previous study [19], three outdoor play categories were established: < 1 h (low outdoor play), 1–2 h (medium outdoor play), and ≥ 2 h (high outdoor play).

#### Statistical analysis

We examined metric variables regarding normal distribution. Skewness values > 2 and kurtosis values > 7 were considered as substantial deviation of normality [32]. To assess retention bias, we compared study completers to dropouts using chi-square and independent sample t-test. Outliers were defined as values with plus/minus three standard deviations around the mean [33]. Analysis was conducted with and without outliers. Significance was set to *P* < 0.05.

In multiple linear regression models, outdoor play was entered as predictor of accelerometer-measured sedentary behavior and moderate-to-vigorous physical activity. As only 1.35% of the included variables had missing data, and this appeared at random, and to use the data that was available, we applied pairwise deletion in the regression model. To set up the regression models, first, we assessed multicollinearity through Pearson correlation analysis [34]. Second, we visually examined residual scatterplots to examine homoscedasticity [34]. Third, we conducted multiple linear regression analysis with outdoor play predicting sedentary behavior and moderate-to-vigorous physical activity in SPSS (version 26). Models were adjusted for sex, age, BMI, and socio-economic status. Fourth, we tested interactions between outdoor play and age due to the wide age range in our study (six to 17 years) and interactions between outdoor play and sex as previous studies indicated differences between boys and girls regarding associations between outdoor play, moderate-to-vigorous physical activity, and sedentary behavior [18–20]. Interactions were tested using PROCESS 3.4 in SPSS [35].

### Study 2

#### Procedures

The participants of Study 2 participated in all three measurement timepoints (T1-T3). The same procedures as described in Study 1 were implemented at all three measurement time points.

#### Participants

For Study 2, participants had to be younger than 18 years at T3 and have participated in all three measurement occasions. A total of 1484 children at T1 were eligible for our analysis, 914 dropped out (reasons unknown due to data protection regulations), resulting in 570 participants (54.7% female; 78.3% normal weight and 9.7% overweight or obese; 62.2% middle and 29.9% high socio-economic status; see Table 2; mean age in years: T1: 5.31 [SD = 0.80]; T2: 11.58 [SD = 0.85]; T3: 16.54 [SD = 0.83]). Study completers were slightly younger, more likely to be female, more likely to have a high socio-economic status, and less likely to be obese. A detailed description of the differences between study completer and non-completers is available in the *Additional File (A3)*.

**Table 2:** Socio-demographic characteristics and weight status of participants in Study 2 at baseline (*N* = 570)

	%	N	% missing
*Sex*			0.0
Female	54.7	312	
Male	45.3	258	
*BMI*			0.4
Underweight	12.0	68	
Normal	78.3	445	
Overweight	8.3	47	
Obese	1.4	8	
*Socio-economic status*			0.2
Low	7.9	45	
Middle	62.2	354	
High	29.9	170	

Abbreviation: BMI = body-mass-index

#### Measures

##### Socio-demographic variables and weight status

The same variables as described in Study 1 were assessed in Study 2 at baseline (T1).

##### Physical activity

Moderate-to-vigorous physical activity was assessed based on the moderate-to-vigorous physical activity index (reported minutes/week) of the MoMo physical activity questionnaire, including school, leisure, and sports clubs moderate-to-vigorous physical activity [16]. It has shown acceptable validity and one-week test-retest reliability (*ICC* = 0.68) [36].

##### Screen-time sedentary behavior

Participants reported their screen-time sedentary behavior (TV/video watching and PC/Gaming time) in minutes per day. Screen-time sedentary behavior has similar directions with health outcomes as direct sedentary behavior measures [37]. Due to previous results [38], we investigated TV watching and PC/Gaming behavior separately, reported as minutes per day. Similar items were used in another study, which reported acceptable reliability and validity for those items (*ICC* = 0.60–0.75; *K* = 0.54–0.69) [39].

##### Outdoor play

Outdoor play was assessed as the number of days with outdoor play, such as playing tag or rope skipping, in a typical week. Participants reported how many days they spent usually playing outside during the week. Response options ranged from 0: “Never” to 7: “daily”. In contrast to Study 1, we could not conduct an analysis based on hours/day as this was not assessed at all three occasions.

#### Statistical analysis

We examined metric variables regarding normal distribution. Skewness values > 2 and kurtosis values > 7 were considered as substantial deviation of normality [32], with variables demonstrating this deviation being transformed [34]. To assess retention bias, we compared study completers to dropouts using chi-square and independent sample t-test. Outliers were defined as values with plus/minus three standard deviations around the mean [33]. Analysis was conducted with and without them. Significance was set to *P* < 0.05.

We used a cross-lag panel design to examine longitudinal associations between the variables of interest [40]. The path panel prediction model was set up using AMOS version 25 with T1, T2, and T3 variables of TV watching and PC/Gaming as screen-time sedentary behaviors, moderate-to-vigorous physical activity, and outdoor play. Stability coefficients were added between same behaviors and cross-lags between different behaviors to obtain a comprehensive picture. However, the cross-lags between moderate-to-vigorous physical activity and screen-time sedentary behaviors have already been reported elsewhere [5], thus, the focus in this study was on the relations between outdoor play, screen-time sedentary behavior, and moderate-to-vigorous physical activity. On average, only 1.94% of the included variables had missing data (see Table 6). To handle missing data, full information maximum likelihood (FIML) was applied in AMOS which shows accurate parameter estimates and fit indices with up to 25% missing data [41]. Model fit was evaluated using the comparative fit index (CFI; values ≥0.90 indicating a good fit), and the root mean square error of approximation (RMSEA; values ≤0.05 indicating a good model fit) [42]. Due to previous results about differences between boys and girls regarding associations between outdoor play, moderate-to-vigorous physical activity, and sedentary behavior [18, 19], we ran the analysis again separately for males and females.

## Results

### Study 1

Participants engaged on average in 554.72 [SD = 122.73] minutes sedentary behavior and 51.16 [SD = 23.58] minutes moderate-to-vigorous physical activity per day. Of the participants, 39.9%, 33.2%, and 26.9% showed low, medium, and high levels of outdoor play, respectively (see Table 3).

**Table 3:** Descriptive statistics of the study variables in Study 1 (N = 2278)

	M	SD	N	%missing
Sedentary behavior (min/day)	554.72	122.73	2278	0.0
Moderate-to-vigorous physical activity (min/day)	51.16	23.58	2278	0.0
Outdoor play			2180	4.3
Low (< 1 h day)			870 (39.9%)	
Medium (1–2 h/day)			723 (33.2%)	
High (≥2 h/day)			587 (26.9%)	

Abbreviations: M = mean, SD = standard deviation, min = minutes

Correlation results revealed no multicollinearity problems. By visual examination, homoscedasticity was confirmed. Results remained similar when outliers were excluded for both analyses, thus we kept outliers in the final models.

The adjusted model explained 63% of variance in sedentary behavior (R^2^ = 0.63; F [9;2094] = 393.31; *P* < 0.001). The model showed that 1–2 h outdoor play was related to 9.75 min and ≥ 2 h outdoor play was related to 17.78 min less sedentary behavior a day compared to < 1 h of outdoor play (see Table 4). With each year older, daily sedentary behavior increased by 28.32 min. Females engaged in 18.06 min more sedentary behavior than boys. No statistically significant interactions between outdoor play and sex or outdoor play and age were observed (see *Additional File A2*).

**Table 4:** Study 1 – Multiple linear regression model predicting sedentary behavior (minutes/day)

	Unstandardized beta	Standard Error	Standardized beta	P	95%-CI
*Model 1*					
Intercept	611.19	3.84		< 0.001	603.66; 618.72
Outdoor play					
Low (< 1 h day)	0 (Ref)				
Medium (1–2 h/day)	−81.37	5.70	−0.31	< 0.001	−92.55; −70.19
High (≥2 h/day)	−109.48	6.05	−0.40	< 0.001	− 121.35; − 97.61
*Model 2*					
Intercept	199.07	10.56		< 0.001	178.36; 219.78
Outdoor play					
Low (< 1 h day)	0 (Ref)				
Medium (1–2 h/day)	−9.75	4.10	−0.04	0.017	−17.78; − 1.72
High (≥2 h/day)	−17.78	4.50	−0.06	< 0.001	− 26.60; − 8.97
Age (years)	28.32	0.55	0.76	< 0.001	27.24; 29.40
*Sex*					
Male	0 (Ref)				
Female	18.06	3.31	0.07	< 0.001	11.56; 24.56
*BMI*					
Normal weight	0 (Ref)				
Underweight	2.95	5.72	0.01	0.606	−8.27; 14.17
Overweight	−4.87	4.93	−0.01	0.323	−14.52; 4.80
Obese	−8.32	8.64	−0.01	0.336	−25.25; 8.62
*Socio-economic status*					
Low	0 (Ref)				
Middle	−0.78	6.35	− 0.003	0.902	−13.24; 11.68
High	2.28	6.86	0.01	0.740	−11.16; 15.72

Model 1: Unadjusted model; Model 2: Adjusted model with the socio-demographic predictors age, sex, BMI, and socio-economic status. Abbreviations: CI = confidence interval; BMI = body mass index

The adjusted model explained 29% of the variance in moderate-to-vigorous physical activity (R^2^ = 0.29; F [9;2094] = 93.21; *P* < 0.001). The model showed that ≥2 h outdoor play was associated with 3.87 min more moderate-to-vigorous physical activity per day (see Table 5). With each one-year older, children had lower moderate-to-vigorous physical activity by 3.08 min. Females had 10.85 min less moderate-to-vigorous physical activity than boys. Children with overweight engaged in 2.96 min less and children with obesity in 5.31 min less moderate-to-vigorous physical activity daily compared to children with normal weight. Children with a high socio-economic status engaged in 3.62 min more moderate-to-vigorous physical activity than children with a low socio-economic status. Outdoor play and sex interacted (*P* < 0.05), indicating that males increased their moderate-to-vigorous physical activity when engaging in outdoor play, while females stayed constant. Also, outdoor play and age interacted (P < 0.05), indicating that until around 15 years, outdoor play engagement was associated with higher moderate-to-vigorous physical activity, but not in 16- and 17-year-olds (see *Additional File, A2*).

**Table 5:** Study 1 – Multiple linear regression model predicting moderate-to-vigorous physical activity (minutes/day)

	Unstandardized beta	Standard Error	Standardized beta	***P***	95%-CI
*Model 1*					
Intercept	43.95	0.77		< 0.001	42.44; 45.46
Outdoor play					
Low (< 1 h day)	0 (Ref)				
Medium (1–2 h/day)	9.81	1.14	0.20	< 0.001	7.56; 12.06
High (≥2 h/day)	14.70	1.22	0.27	< 0.001	12.32; 17.09
*Model 2*					
Intercept	93.07	2.81		< 0.001	87.55; 98.59
Outdoor play					
Low (< 1 h day)	0 (Ref.)				
Medium (1–2 h/day)	0.85	1.09	0.02	0.434	−1.29; 2.99
High (≥2 h/day)	3.87	1.20	0.07	0.001	1.53; 6.22
Age (years)	−3.08	0.15	−0.43	< 0.001	−3.37; −2.79
*Sex*					
Male	0 (Ref.)				
Female	−10.85	0.88	−0.23	< 0.001	− 12.58; −9.11
*BMI*					
Normal weight	0 (Ref.)				
Underweight	−0.50	1.52	−0.01	0.744	−3.49; 2.49
Overweight	−2.96	1.31	−0.04	0.024	−5.53; −0.38
Obese	−5.31	2.30	−0.04	0.021	−9.82; −0.80
*Socio-economic status*					
Low	0 (Ref.)				
Middle	1.10	1.69	0.02	0.514	−2.22; 4.42
High	3.62	1.83	0.07	0.047	0.04; 7.20

Model 1: Unadjusted model; Model 2: Adjusted model with the socio-demographic predictors age, sex, BMI, and socio-economic status. Abbreviations: CI = confidence interval; BMI = body mass index

### Study 2

Participants engaged in 142.09 [SD = 119.24] minutes moderate-to-vigorous physical activity per week at T1, which increased to 304.82 [SD = 182.22] minutes per week at T2, before dropping to 262.74 [SD = 208.60] minutes per week at T3. TV watching consistently increased from 54.77 [SD = 37.96] minutes per day at T1 to 127.49 [SD = 89.25] minutes per day at T3. PC/Gaming increased from 5.77 [SD = 14.09] minutes per day at T1 to 187.42 [SD = 137.94] minutes per day at T3. Outdoor play decreased from 5.93 [SD = 1.43] days per week at T1 to 1.14 [SD = 1.85] days per week at T3 (see Table 6).

**Table 6:** Descriptive statistics of the study variables in Study 2 (N = 570)

	M	SD	N	%missing
Moderate-to-vigorous physical activity T1 (min/week)	142.09	119.24	570	0.0
Outdoor play T1 (days/week)	5.93	1.43	556	2.4
TV/Video watching T1 (min/day)	54.77	37.96	568	0.3
PC/Gaming time T1 (min/day)	5.77	14.09	556	2.4
Moderate-to-vigorous physical activity T2 (min/week)	304.82	183.22	570	0.0
Outdoor play T2 (days/week)	4.42	2.14	566	0.7
TV/Video watching T2 (min/day)	69.52	56.38	564	1.1
PC/Gaming timeT2 (min/day)	60.43	62.13	560	1.7
Moderate-to-vigorous physical activity T3 (min/week)	262.74	208.60	560	1.7
Outdoor play T3 (days/week)	1.14	1.65	513	10.0
TV/Video watching T3 (min/day)	127.49	89.25	565	0.9
PC/Gaming timeT3 (min/day)	187.42	137.94	558	2.1

Abbreviations: M = mean, SD = standard deviation, min = minutes

As PC/Gaming_T1_, PC/Gaming_T2_, and moderate-to-vigorous physical activity_T2_ showed skewness or kurtosis values above the accepted range, we used square-root transformation to normalize the data [34]. We ran the analysis with outliers excluded which did not change the results, thus, they were included in the final model.

**Fig. 1:**
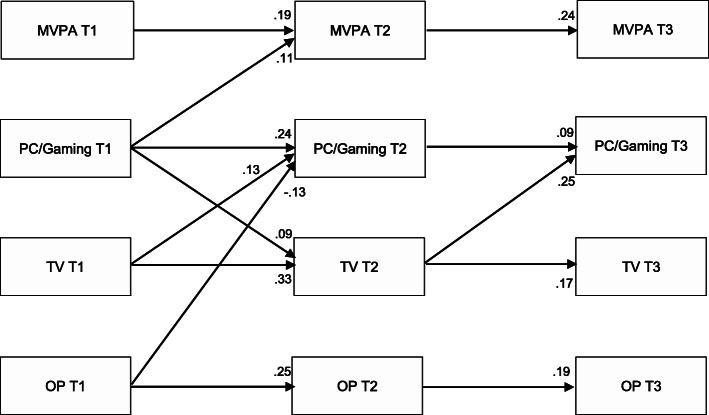
Study 2 – Cross-lag panel model between outdoor play, screen-time sedentary behavior, and moderate-to-vigorous physical activity. OP = outdoor play, MVPA = moderate to vigorous physical activity. Please note: Only associations *P* < 0.05 are reported in the figure. All coefficients are standardized estimates

The path prediction model showed a good model fit (CFI = 0.949; RMSEA = 0.047 [CI-90% = 0.029–0.065]). We only report the results *P* < 0.05 as standardized estimates (see Fig. 1), all estimates are reported in the *Additional file (A4)*. For screen-time sedentary behavior and outdoor play, stability coefficients were stronger from early to later childhood, whereas moderate-to-vigorous physical activity was stronger from later childhood to adolescence (see Fig. 1). Regarding the cross-behavior relationships, prospective associations were stronger between early and later childhood: outdoor play_T1_ negatively predicted PC/Gaming_T2_ (standardized estimate = − 0.13; *P* < 0.001). TV_T1_ positively predicted PC/Gaming_T2_ (standardized estimate = 0.013; P < 0.001). PC/Gaming_T1_ positively predicted TV_T2_ (standardized estimate = 0.09; *P* = 0.004) and moderate-to-vigorous physical activity_T2_ (standardized estimate = 0.11; *P* = 0.010). From childhood to adolescence, only TV_T2_ positively predicted PC/Gaming_T3_ (standardized estimate = 0.25; P < 0.001). The model explained 12.9% of the variance in TV_T2_, 11.8% in PC/Gaming_T2_, 5.5% in moderate-to-vigorous physical activity_T2_, 2.7% in TV_T3_, 8.7% in PC/Gaming_T3_, and 6.6% in moderate-to-vigorous physical activity_T3_. We re-ran the analysis separately for males and females, with the results being the same for boys and girls, thus, only the overall model is reported.

## Discussion

The aim of this study was to investigate cross-sectional and prospective associations between outdoor play, sedentary behavior (screen-time), and moderate-to-vigorous physical activity.

Study 1 showed that engagement in outdoor play was associated with lower accelerometer-measured sedentary behavior, which is supported by previous findings that used device-based assessment of sedentary behavior [19]. Regarding socio-demographic predictors, older age was the strongest predictor of sedentary behavior among youth. In our sample, each one-year increase in age was associated with about half an hour-increase in sedentary behavior per day. A similar strong result has been observed in a previous study with nine- to 11-year-old children, with a one-year increase being associated with a 26-min increase in sedentary behavior per day [43]. Also, other studies have consistently reported positive associations between sedentary behavior and children’s age [9, 44]. The strong increase in sedentary behavior may be explained via more study time in higher school grades [45], more sedentary behavior in school [46], as well as more leisure-time engagement in screen-time behaviors such as gaming and recreational internet use [11]. Also, our results indicate that girls spend more time in sedentary behavior than boys, which is in line with previous findings [47]. A reason for this could be that girls engage in various types of sedentary behavior that go beyond screen-time sedentary behavior, such as homework and talking on the phone whilst being similar to boys in screen-time sedentary behavior [48], resulting in more total sedentary behavior. Interestingly, in contrast to physical activity, there was no interaction observed with age, which means that at any age between six and 17 years, more outdoor play is associated with lower sedentary behavior. A reason for this could be that older children and adolescents still engage in active types of outdoor play, which may not be intense enough to translate into moderate-to-vigorous physical activity, but still be more active than sedentary behavior (e.g., playing at a swimming pool, meeting for a round of badminton).

Regarding moderate-to-vigorous physical activity, having a normal weight and a high socio-economic status were positively related to moderate-to-vigorous physical activity, whereas being a girl was negatively related to moderate-to-vigorous physical activity, which is in line with previous findings [49, 50]. The gender gap observed in physical activity in our data is comparable to other high-income Western countries in Europe [51]. The reasons why girls have lower physical activity levels in general are complex, including less enjoyment and confidence in their sporting abilities, gender norms [52], stronger perceived physical activity barriers in girls, such as lack of time, social influence, and willpower [53], as well as limited independent mobility of girls compared to boys [54]. Engagement in ≥2 h of outdoor play was associated with increased moderate-to-vigorous physical activity, with this relationship being moderated by sex, showing that more outdoor play is only related to more moderate-to-vigorous physical activity in boys. This is similar to findings in previous studies, showing that outdoor play is mostly unrelated to moderate-to-vigorous physical activity in girls [19, 20]. One explanation could be that girls in Germany engage in less active types of outdoor play than boys [55], which might result in lower activity intensities that do not add to moderate-to-vigorous physical activity. Another reason could be that boys across countries are allowed to play outdoors with less supervision and with a larger spatial range from the residential home [56], resulting in more outdoor play opportunities.

The association between outdoor play and moderate-to-vigorous physical activity was also moderated by age, indicating that outdoor play was only related to increased moderate-to-vigorous physical activity until around 15 years. One reason could be that children engage in more intense types of outdoor play, such as rope skipping, running around in the garden, or playing games such as tag with other children. In contrast, adolescents may engage in less intense types of outdoor play, such as passing a ball to each other. However, to investigate the underlying mechanisms for this interaction, a more specific analysis of outdoor play would be necessary to investigate in which types of outdoor play children and adolescents engage at which age and how the different types relate to moderate-to-vigorous physical activity [18].

Study 2 revealed that outdoor play predicts PC/Gaming as one type of screen-time sedentary behavior from early to later childhood, but not TV watching or moderate-to-vigorous physical activity. No other longitudinal studies were found that investigated outdoor play, and screen-time sedentary behavior across childhood and adolescence, while cross-sectional findings to date showed largely no relationship between screen time or exposure and outdoor play [18]. A reason why outdoor play negatively predicts PC/Gaming but not TV watching could be that outdoor play is an active behavior [12], while PC/Gaming has also been categorized as an active (sedentary) behavior as it requires mentally active engagement [57]. Contrary, TV watching is categorized as a passive sedentary behavior [57]. Thus, if children develop outdoor play as an active behavior in young childhood, they might become less involved in screen-based active behaviors in later childhood, while TV watching as a passive behavior seems to be independent of outdoor play. Hence, outdoor play may be a leisure pursuit that can replace computer and gaming time. When considering outdoor play as a replacement for sedentary behavior, it would be valuable to investigate the type of outdoor play that can replace sedentary behavior in the future. This is especially relevant as replacing sedentary time with some physical activity, including light-intensity physical activity as we may see in older children’s and adolescent’s outdoor play, show favorable associations with cardiometabolic risk factors, such as waist circumference [58], as well as with mental health outcomes [59]. No prospective relationship was found between outdoor play and PC/Gaming from late childhood to adolescence. Although the stability coefficient indicated that children’s outdoor play behavior tracks to adolescence, outdoor play decreases in general with age. Thus, in adolescence, outdoor play might be independent of sedentary behavior and moderate-to-vigorous physical activity.

Regarding outdoor play and moderate-to-vigorous physical activity, we are not aware of another study that investigated prospective relationships between the two behaviors during childhood and adolescence. A long-term follow-up study that investigated relationships between childhood outdoor play and sports or physical activity participation in adulthood supports the results of this study [21]. Outdoor play consists of various activities, however not every activity is conducted with a health-enhancing intensity. In fact, one analysis showed that preschool children spent about half of their outdoor play in physical activity, but only 14% in moderate-to-vigorous physical activity [17], indicating that outdoor play rather relates to light intensity physical activity. Another reason could be that outdoor play and moderate-to-vigorous physical activity should be assessed more specifically as they both comprise various activities.

As stated, all cross-lags not related to outdoor play have already been investigated in this sample, thus, we refer to Nigg et al. [5] for a discussion of those results.

Considering results of Study 1 and Study 2, both studies support a relationship between outdoor play and sedentary behavior (screen-time) and that this relationship is present for boys and girls. Hence, outdoor play may be a behavior across the developmental stage of childhood and adolescence that has the potential to replace sedentary behavior and thus promote both physical and mental health in children and adolescents [58, 59]. Regarding moderate-to-vigorous physical activity, Study 1 and Study 2 had inconsistent results: While Study 1 supported a relationship between outdoor play and moderate-to-vigorous physical activity, no longitudinal associations were observed in Study 2. A reason for this could be the focus on sports-related moderate-to-vigorous physical activity in the self-reported data, which neglects moderate-to-vigorous physical activity in daily life (Study 2), while daily life moderate-to-vigorous physical activity is captured through accelerometer-measurement, which might be more relevant for outdoor play (Study 1). Another reason could be that the assessment of outdoor play in days per week (Study 2) is not sensitive enough to detect associations compared to hours per day (Study 1). Thus, future studies should investigate longitudinal associations between the three behaviors using more fine-grained outdoor play operationalization and device-based assessment of moderate-to-vigorous physical activity and sedentary behavior.

### Limitations and strengths

There are some limitations to be considered. In Study 2, data is based on self-report. Temporal order of variables could be established but not causality [34, 40] and the duration between assessments was long. Only PC/Gaming time along with TV watching were assessed for sedentary behavior, not accounting for other non-screen-based sedentary behavior, e.g., homework or reading. For both Study 1 and Study 2, assessment for one measurement timepoint across the whole year, which may lead to seasonality effects; however, the MoMo physical activity questionnaire specifically asks for activity types across all months to counteract those effects. Finally, differences between study completers and non-completers may have influenced the associations investigated.

Nonetheless, to our best knowledge, this is one of the first studies that investigated prospective associations between children’s and adolescent’s outdoor play, sedentary behavior and moderate-to-vigorous physical across more than a decade. This allowed to explore potential relationships regarding the temporal order of the variables, as to date, mostly cross-sectional studies are available [60]. Also, to our best knowledge, no studies explored the relationship between outdoor play and accelerometer-measured moderate-to-vigorous physical activity and sedentary behavior in a large sample of children and adolescents in Germany across a broad age range, with this studies’ results mirroring results from previous studies in other countries [19, 21, 60].

## Conclusion

Outdoor play during early childhood negatively predicted PC/Gaming time in later childhood, while there was no longitudinal association with moderate-to-vigorous physical activity. Results of the cross-sectional analysis indicated that engagement in outdoor play was related to lower sedentary behavior independent of socio-demographic characteristics, whereas for moderate-to-vigorous physical activity, the relationship was dependent on sex and age. Practically speaking, increasing outdoor play time in preschool children might be one way to prevent PC/Gaming time in later childhood. Thus, early childhood settings should provide outdoor play opportunities. Future research should investigate prospective relationships between the three behaviors using device-based assessments, with a special focus on investigating which types of outdoor play are relevant to replace sedentary behavior and promote physical activity.

## Supplementary Information

**Additional file 1.** Includes comparison between study completers and non-completers, regressions weights of the path panel prediction model, and the models with the interactions of the cross-sectional analysis.

## Data Availability

Due to federal data protection law, it is not possible to make this data publicly available. The datasets used and/or analyzed during the current study are available from the corresponding author on reasonable request.

## Abbreviations

BMI: body-mass-index
SB: sedentary behavior
MVPA: moderate-to-vigorous physical activity

## References

[1] Carson V, Hunter S, Kuzik N, Gray CE, Poitras VJ, Chaput J-P (2016). Systematic review of sedentary behaviour and health indicators in school-aged children and youth: an update. Appl Physiol Nutr Metab.

[2] Janssen I, Leblanc AG (2010). Systematic review of the health benefits of physical activity and fitness in school-aged children and youth. Int J Behav Nutr Phys Act.

[3] Hinkley T, Brown H, Carson V, Teychenne M (2018). Cross sectional associations of screen time and outdoor play with social skills in preschool children. PLoS One.

[4] Li J, Hestenes LL, Wang YC (2016). Links between preschool Children’s social skills and observed pretend play in outdoor childcare environments. Early Childhood Educ J.

[5] Nigg CR, Wunsch K, Nigg C, Niessner C, Jekauc D, Schmidt SCE (2021). Is Physical Activity, Screen Time, and Mental Health Related during Childhood, Preadolescence, and Adolescence? 11-Year Results from the German MoMo Cohort Trial. Am J Epidemiol.

[6] Tremblay MS, Aubert S, Barnes JD, Saunders TJ, Carson V, Latimer-Cheung AE, et al. Sedentary Behavior Research Network (SBRN) – Terminology Consensus Project process and outcome. Int J Behav Nutr Phys Act. 2017;14:75. 10.1186/s12966-017-0525-8. 10.1186/s12966-017-0525-8 PMC5466781 28599680

[7] Caspersen CJ, Powell KE, Christenson GM (1985). Physical activity, exercise, and physical fitness: definitions and distinctions for health-related research. Public Health Rep.

[8] WHO. WHO Guidelines on physical activity and sedentary behaviour. Geneva, Switzerland: World Health Organization; 2020.

[9] Pate RR, Mitchell JA, Byun W, Dowda M (2011). Sedentary behaviour in youth. Br J Sports Med.

[10] Sallis JF, Bull F, Guthold R, Heath GW, Inoue S, Kelly P, Oyeyemi AL, Perez LG, Richards J, Hallal PC (2016). Progress in physical activity over the Olympic quadrennium. Lancet.

[11] Schmidt SCE, Anedda B, Burchartz A, Eichsteller A, Kolb S, Nigg C, et al. Physical activity and screen time of children and adolescents before and during the COVID-19 lockdown in Germany: a natural experiment. Sci Rep. 2020;10:21780. 10.1038/s41598-020-78438-4. 10.1038/s41598-020-78438-4 PMC7733438 33311526

[12] Tremblay M, Gray C, Babcock S, Barnes J, Bradstreet C, Carr D, Chabot G, Choquette L, Chorney D, Collyer C, Herrington S, Janson K, Janssen I, Larouche R, Pickett W, Power M, Sandseter E, Simon B, Brussoni M (2015). Position statement on active outdoor play. Int J Environ Res Public Health.

[13] Ansari A, Pettit K, Gershoff E (2015). Combating obesity in head start: outdoor play and change in Children's body mass index. J Dev Behav Pediatr.

[14] Janssen I (2016). Estimating whether replacing time in active outdoor play and sedentary video games with active video games influences Youth's mental health. J Adolesc Health.

[15] Piccininni C, Michaelson V, Janssen I, Pickett W (2018). Outdoor play and nature connectedness as potential correlates of internalized mental health symptoms among Canadian adolescents. Prev Med.

[16] Schmidt SCE, Anedda B, Burchartz A, Oriwol D, Kolb S, Wäsche H, Niessner C, Woll A (2020). The physical activity of children and adolescents in Germany 2003-2017: the MoMo-study. PLoS One.

[17] Truelove S, Bruijns BA, Vanderloo LM, O'Brien KT, Johnson AM, Tucker P. Physical activity and sedentary time during childcare outdoor play sessions: a systematic review and meta-analysis. Prev Med. 2018;108:74–85. 10.1016/j.ypmed.2017.12.022. 10.1016/j.ypmed.2017.12.022 29305869

[18] Lee E-Y, Bains A, Hunter S, Ament A, Brazo-Sayavera J, Carson V, et al. Systematic review of the correlates of outdoor play and time among children aged 3-12 years. Int J Behav Nutr Phys Act. 2021;18:41. 10.1186/s12966-021-01097-9. 10.1186/s12966-021-01097-9 PMC7972019 33736668

[19] Stone MR, Faulkner GEJ (2014). Outdoor play in children: associations with objectively-measured physical activity, sedentary behavior and weight status. Prev Med.

[20] Appelhans BM, Li H (2016). Organized sports and unstructured active play as physical activity sources in children from low-income Chicago households. Pediatr Exerc Sci.

[21] Smith L, Gardner B, Aggio D, Hamer M (2015). Association between participation in outdoor play and sport at 10years old with physical activity in adulthood. Prev Med.

[22] Vandenbroucke JP, Von Elm E, Altman DG, Gøtzsche PC, Mulrow CD, Pocock SJ (2007). Strengthening the reporting of observational studies in epidemiology (STROBE). Epidemiology..

[23] Woll A, Albrecht C, Worth A (2017). Motorik-Module (MoMo) – the KiGGS Wave 2 module to survey motor performance and physical activity. J Health Monitor.

[24] Kurth B-M, Kamtsiuris P, Hölling H, Schlaud M, Dölle R, Ellert U, Kahl H, Knopf H, Lange M, Mensink GBM, Neuhauser H, Rosario AS, Scheidt-Nave C, Schenk L, Schlack R, Stolzenberg H, Thamm M, Thierfelder W, Wolf U (2008). The challenge of comprehensively mapping children's health in a nation-wide health survey: design of the German KiGGS-study. BMC Public Health.

[25] Kamtsiuris P, Lange M, Schaffrath AR (2007). The German health interview and examination survey for children and adolescents (KiGGS): sample design, response and nonresponse analysis. Bundesgesundheitsblatt, Gesundheitsforschung, Gesundheitsschutz.

[26] Lampert T, Müters S, Stolzenberg H, Kroll LE (2014). Messung des sozioökonomischen status in der KiGGS-Studie [assessment of the socio-economic status in the KiGGS-study]. Bundesgesundheitsblatt, Gesundheitsforschung, Gesundheitsschutz..

[27] Cole TJ, Bellizzi MC, Flegal KM, Dietz WH (2000). Establishing a standard definition for child overweight and obesity worldwide: international survey. BMJ.

[28] Burchartz A, Manz K, Anedda B, Niessner C, Oriwol D, Schmidt S (2020). Measurement of physical activity and sedentary behavior by accelerometry among a nationwide sample of the KiGGS and MoMo study: a study protocol. JMIR Res Protocols.

[29] Choi L, Liu Z, Matthews CE, Buchowski MS (2011). Validation of accelerometer wear and nonwear time classification algorithm. Med Sci Sports Exerc.

[30] Evenson KR, Catellier DJ, Gill K, Ondrak KS, McMurray RG (2008). Calibration of two objective measures of physical activity for children. J Sports Sci.

[31] Romanzini M, Petroski EL, Ohara D, Dourado AC, Reichert FF (2014). Calibration of ActiGraph GT3X, Actical and RT3 accelerometers in adolescents. Eur J Sport Sci.

[32] Kim H-Y (2013). Statistical notes for clinical researchers: assessing normal distribution (2) using skewness and kurtosis. Restor Dent Endod.

[33] Howell D (1998). Statistical methods in human sciences.

[34] Tabachnick BG, Fidell LS (2013). Using multivariate statistics.

[35] Hayes AF (2018). Introduction to mediation, moderation, and conditional process analysis: a regression-based approach.

[36] Jekauc D, Voelkle M, Wagner MO, Mewes N, Woll A (2012). Reliability, validity, and measurement invariance of the German version of the physical activity enjoyment scale. J Pediatr Psychol.

[37] Tremblay MS, Leblanc AG, Kho ME, Saunders TJ, Larouche R, Colley RC (2011). Systematic review of sedentary behaviour and health indicators in school-aged children and youth. Int J Behav Nutr Phys Act.

[38] Mathers M, Canterford L, Olds T, Hesketh K, Ridley K, Wake M (2009). Electronic media use and adolescent health and well-being: cross-sectional community study. Acad Pediatr.

[39] Cabanas-Sánchez V, Martínez-Gómez D, Esteban-Cornejo I, Castro-Piñero J, Conde-Caveda J, Veiga ÓL (2018). Reliability and validity of the youth leisure-time sedentary behavior questionnaire (YLSBQ). J Sci Med Sport.

[40] Selig JP, Little TD, Laursen B, Little TD, Card NA (2012). Autoregressive and cross-lagged panel analysis for longitudinal data. Handbook of developmental research methods.

[41] Enders C, Bandalos D (2001). The relative performance of full information maximum likelihood estimation for missing data in structural equation models. Struct Equ Modeling..

[42] Hu LT, Bentler PM (1999). Cutoff criteria for fit indexes in covariance structure analysis: conventional criteria versus new alternatives. Struct Equ Modeling.

[43] Farías NA, Fuentealba PM, Poblete DC. Correlates of device-measured physical activity, sedentary behaviour and sleeping in children aged 9–11 years from Chile: ESPACIOS study. Retos. 2020;(37):1–10.

[44] Vancampfort D, Van Damme T, Firth J, Hallgren M, Smith L, Stubbs B (2019). Correlates of leisure-time sedentary behavior among 181,793 adolescents aged 12-15 years from 66 low- and middle-income countries. PLoS One.

[45] Cheema JR, Sheridan K (2015). Time spent on homework, mathematics anxiety and mathematics achievement: evidence from a US sample. Issues Educ Res.

[46] Mooses K, Mägi K, Riso E-M, Kalma M, Kaasik P, Kull M. Objectively measured sedentary behaviour and moderate and vigorous physical activity in different school subjects: a cross-sectional study. BMC Public Health. 2017;17:108. 10.1186/s12889-017-4046-9 10.1186/s12889-017-4046-9 PMC5260134 28114919

[47] Bailey DP, Fairclough SJ, Savory LA, Denton SJ, Pang D, Deane CS, Kerr CJ (2012). Accelerometry-assessed sedentary behaviour and physical activity levels during the segmented school day in 10–14-year-old children: the HAPPY study. Eur J Pediatr.

[48] Taverno Ross SE, Byun W, Dowda M, McIver KL, Saunders RP, Pate RR (2013). Sedentary behaviors in fifth-grade boys and girls: where, with whom, and why?. Child Obes.

[49] Janssen I, Katzmarzyk PT, Boyce WF, Vereecken C, Mulvihill C, Roberts C, Currie C, Pickett W, The Health Behaviour in School-Aged Children Obesity Working Group* (2005). Comparison of overweight and obesity prevalence in school-aged youth from 34 countries and their relationships with physical activity and dietary patterns. Obes Rev.

[50] Lämmle L, Worth A, Bos K (2012). Socio-demographic correlates of physical activity and physical fitness in German children and adolescents. Eur J Pub Health.

[51] Guthold R, Stevens GA, Riley LM, Bull FC (2020). Global trends in insufficient physical activity among adolescents: a pooled analysis of 298 population-based surveys with 1·6 million participants. Lancet Child Adolesc Health.

[52] The Lancet Public H (2019). Time to tackle the physical activity gender gap. Lancet Public Health.

[53] Rosselli M, Ermini E, Tosi B, Boddi M, Stefani L, Toncelli L, Modesti PA (2020). Gender differences in barriers to physical activity among adolescents. Nutr Metab Cardiovasc Dis.

[54] Schoeppe S, Duncan MJ, Badland HM, Rebar AL, Vandelanotte C (2016). Too far from home? Adult attitudes on children's independent mobility range. Children's Geographies.

[55] Reimers A, Schoeppe S, Demetriou Y, Knapp G (2018). Physical activity and outdoor play of children in public playgrounds—do gender and social environment matter?. Int J Environ Res Public Health.

[56] Lee H, Tamminen KA, Clark AM, Slater L, Spence JC, Holt NL (2015). A meta-study of qualitative research examining determinants of children’s independent active free play. Int J Behav Nutr Phys Act.

[57] Hallgren M, Dunstan DW, Owen N (2020). Passive versus mentally active sedentary behaviors and depression. Exerc Sport Sci Rev.

[58] Hansen BH, Anderssen SA, Andersen LB, Hildebrand M, Kolle E, Steene-Johannessen J (2018). Cross-sectional associations of reallocating time between sedentary and active Behaviours on Cardiometabolic risk factors in young people: an international Children’s Accelerometry database (ICAD) analysis. Sports Med.

[59] Kandola A, Lewis G, Osborn DPJ, Stubbs B, Hayes JF (2020). Depressive symptoms and objectively measured physical activity and sedentary behaviour throughout adolescence: a prospective cohort study. Lancet Psychiatry.

[60] Brussoni M, Gibbons R, Gray C, Ishikawa T, Sandseter E, Bienenstock A, Chabot G, Fuselli P, Herrington S, Janssen I, Pickett W, Power M, Stanger N, Sampson M, Tremblay M (2015). What is the relationship between risky outdoor play and health in children? A systematic review. Int J Environ Res Public Health.

