# Supplementary material for: Relating outdoor play to sedentary behavior and physical activity in youth - results from a cohort study
Source: BMC Public Health. 2021 Sep 21;21:1716. doi: 10.1186/s12889-021-11754-0 (PMC8456698; doi:10.1186/s12889-021-11754-0)
Supplement: Supplementary file 1 — Additional file 1. Includes comparison between study completers and non-completers, regressions weights of the path panel prediction model, and the models with the interactions of the cross-sectional analysis. [file 12889_2021_11754_MOESM1_ESM.docx]

**Additional File**

**Study 1**

**A1. Differences between study completers and non-completers**

Study completers were more likely to be female (completers: 52.9%; dropout: 48.8%; *V*=0.04; P=0.006). They were slightly younger (completers: 12.52 years [SD = 3.30], dropout: 12.94 years [SD = 3.30]; *V*=0.13; P<0.001) and more likely to have a healthy weight (completers: 73.3%; dropout: 72.0%; *V*=0.06; P=0.004).

**A2. Cross-sectional analysis – models with interactions using PROCESS version 3.4**

*Multiple linear regression model with outdoor play * age interaction predicting sedentary behavior (minutes/day).*

|  | **Unstandardized beta** | **Standard Error** | ***P*** | **95%-CI** |
| --- | --- | --- | --- | --- |
| Intercept | 206.03 | 14.62 | <0.001 | 177.35; 234.71 |
| OP (hours/day) |  |  |  |  |
| Low (< 1 hour day) | 0 (Ref.) |  |  |  |
| Medium (1-2 hours/day) | -34.19 | 17.05 | 0.045 | -67.61; -0.75 |
| High (≥2 hours/day) | -38.53 | 17.32 | 0.026 | -72.48; -4.57 |
| Age | 28.04 | 0.92 | <0.001 | 26.24; 29.84 |
| OP (hours/day)*age |  |  |  |  |
| Low OP (<1) | 0 (Ref) |  |  |  |
| Medium OP (1-2) | 1.81 | 1.30 | 0.164 | -0.74; 4.35 |
| High OP (≥2) | 1.57 | 1.36 | 0.249 | -1.10; 4.24 |
| *Sex* |  |  |  |  |
| Males | 0 (Ref) |  |  |  |
| Females | 17.72 | 3.27 | <0.001 | 11.31; 24.13 |
| *BMI* |  |  |  |  |
| Normal weight | 0 (Ref) |  |  |  |
| Underweight | 3.06 | 5.61 | 0.586 | -7.94; 14.06 |
| Overweight | 0.10 | 4.99 | 0.985 | -9.68; 9.87 |
| Obese | -8.57 | 8.48 | 0.312 | -25.20; 8.06 |
| *Socio-economic status* |  |  |  |  |
| Low | 0 (Ref.) |  |  |  |
| Middle | -0.58 | 6.37 | 0.927 | -13.06; 11.90 |
| High | 2.84 | 6.82 | 0.678 | -10.55; 16.23 |

Abbreviations: CI = confidence interval; OP = outdoor play, BMI = body mass index

*Multiple linear regression model with outdoor play * sex interaction predicting sedentary behavior (minutes/day).*

|  | **Unstandardized beta** | **Standard Error** | ***P*** | **95%-CI** |
| --- | --- | --- | --- | --- |
| Intercept | 188.31 | 10.70 | <0.001 | 167.32; 209.30 |
| OP (hours/day) |  |  |  |  |
| Low (< 1 hour day) | 0 (Ref) |  |  |  |
| Medium (1-2 hours/day) | -9.47 | 5.83 | 0.105 | -20.89; 1.96 |
| High (≥2 hours/day) | -11.79 | 6.14 | 0.055 | -23.84; 0.26 |
| *Sex* |  |  |  |  |
| Males | 0 (Ref) |  |  |  |
| Females | 21.70 | 5.31 | <0.001 | 11.28; 32.11 |
| OP (hours/day)*sex |  |  |  |  |
| Low OP (<1) | 0 (Ref) |  |  |  |
| Medium OP (1-2) | -1.68 | 7.69 | 0.828 | -16.75; 13.41 |
| High OP (≥2) | -12.28 | 8.12 | 0.131 | -28.21; 3.65 |
| Age | 29.11 | 0.55 | <0.001 | 28.03; 30.18 |
| *BMI* |  |  |  |  |
| Normal weight | 0 (Ref) |  |  |  |
| Underweight | 3.42 | 5.61 | 0.542 | -7.58; 14.41 |
| Overweight | 0.22 | 4.98 | 0.964 | -9.55; 10.00 |
| Obese | -8.00 | 8.48 | 0.346 | -24.62; 8.62 |
| *Socio-economic status* |  |  |  |  |
| Low | 0 (Ref) |  |  |  |
| Middle | -0.27 | 3.67 | 0.966 | -12.75; 12.21 |
| High | 3.33 | 6.83 | 0.626 | -10.07; 16.72 |

Abbreviations: CI = confidence interval; OP = outdoor play, BMI = body mass index

*Multiple linear regression model with outdoor play * sex interaction predicting moderate-to-vigorous physical activity (minutes/day).*

|  | **Unstandardized beta** | **Standard Error** | ***P*** | **95%-CI** |
| --- | --- | --- | --- | --- |
| Intercept | 92.02 | 3.24 | <0.001 | 85.67; 98.37 |
| Outdoor play |  |  |  |  |
| Low (< 1 hour day) | 0 (Ref) |  |  |  |
| Medium (1-2 hours/day) | 3.57 | 1.60 | 0.025 | 0.44; 6.69 |
| High (≥2 hours/day) | 6.96 | 1.68 | <0.001 | 3.66; 10.25 |
| *Sex* |  |  |  |  |
| Males | 0 (Ref) |  |  |  |
| Females | -7.94 | 1.45 | <0.001 | -10.79; -5.09 |
| OP (hours/day)*sex |  |  |  |  |
| Low OP (<1) | 0 (Ref) |  |  |  |
| Medium OP (1-2) | -4.75 | 2.10 | 0.024 | -8.87; -0.62 |
| High OP (≥2) | -5.70 | 2.22 | 0.010 | -10.05; -1.34 |
| Age (years) | -3.15 | 0.15 | <0.001 | -3.45; -2.86 |
| *BMI* |  |  |  |  |
| Normal weight | 0 (Ref) |  |  |  |
| Underweight | 0.34 | 1.53 | 0.826 | -2.67; 3.35 |
| Overweight | -3.40 | 1.92 | 0.078 | -7.16; 0.38 |
| Obese | -5.28 | 2.69 | 0.049 | -10.56; -0.02 |
| *Socio-economic status* |  |  |  |  |
| Low | 0 (Ref) |  |  |  |
| Middle | 1.00 | 1.74 | 0.566 | -2.41; 4.41 |
| High | 3.55 | 1.87 | 0.057 | -0.11; 7.22 |

Abbreviations: CI = confidence interval; BMI = body mass index

*Multiple linear regression model with outdoor play * age interaction predicting moderate-to-vigorous physical activity (minutes/day).*

|  | **Unstandardized beta** | **Standard Error** | ***P*** | **95%-CI** |
| --- | --- | --- | --- | --- |
| Intercept | 81.92 | 3.98 | <0.001 | 74.09; 86.73 |
| OP (hours/day) |  |  |  |  |
| Low (< 1 hour day) | 0 (Ref) |  |  |  |
| Medium (1-2 hours/day) | 19.61 | 4.65 | <0.001 | 10.49; 28.72 |
| High (≥2 hours/day) | 20.63 | 4.72 | <0.001 | 11.37; 29.89 |
| Age | -2.27 | 0.25 | <0.001 | -2.76; -1.78 |
| OP (hours/day)*age |  |  |  |  |
| Low OP (<1) | 0 (Ref) |  |  |  |
| Medium OP (1-2) | -1.44 | 0.35 | 0.001 | -2.13; -0.74 |
| High OP (≥2) | -1.29 | 0.37 | 0.005 | -2.02; -0.56 |
| *Sex* |  |  |  |  |
| Males | 0 (Ref) |  |  |  |
| Females | -11.20 | 0.89 | <0.001 | -12.95; -9.45 |
| *BMI* |  |  |  |  |
| Normal weight | 0 (Ref) |  |  |  |
| Underweight | -0.17 | 1.53 | 0.913 | -3.17; 2.83 |
| Overweight | -3.57 | 1.35 | 0.009 | -6.23; -0.90 |
| Obese | -5.36 | 2.31 | 0.021 | -9.90; -0.82 |
| *Socio-economic status* |  |  |  |  |
| Low | 0 (Ref) |  |  |  |
| Middle | 0.91 | 1.74 | 0.601 | -2.50; 4.31 |
| High | 3.61 | 1.86 | 0.053 | -0.04; 7.26 |

Abbreviations: CI = confidence interval; OP = outdoor play, BMI = body mass index

**Study 2**

**A3. Differences between study completers and non-completers**

Study completers were slightly younger (completers: 5.31 years [SD=0.80], dropout: 5.64 years [SD=0.87]; *d*=0.39; P<0.001), more likely to be female (completers: 54.7%; dropout: 47.7%; *V*=0.07; P=0.008), more likely to have a high SES (completers: 29.9%; dropout: 21.3%; *V*=0.16; P<0.001), and less likely to be obese (completers: 1.4%; dropout: 3.9%; *V*=0.08; P=0.035). Study completers watched less TV (completers: 54.77 minutes [SD=37.96], dropout: 68.05 minutes [SD=51.60]; *d*=0.29; P<0.001), had less PC/Gaming time (completers: 5.77 minutes [SD=14.08]; dropout: 8.94 minutes [SD=20.35]; *d*=0.39; P=0.001), and tended to do less MVPA (completers: 142.08 minutes [SD=119.24]; dropout: 154.65 minutes [SD=5.20]; *d*=0.09; P=0.082)

**A4. Longitudinal analysis - Results of the path prediction model**

*Regression weights*

| **Independent variable** | **Dependent variable** | **Unstandardized estimate** | **Standardized estimate** | **Standard Error** | ***P*** | **95%-CI** | |
| --- | --- | --- | --- | --- | --- | --- | --- |
|  |  |  |  |  |  | **Lower** | **Upper** |
| MVPA T1 | MVPA T2 | 0.01 | 0.19 | 0.002 | <0.001 | 0.004 | 0.01 |
| MVPA T1 | OP T2 | <0.001 | -0.02 | 0.001 | 0.59 | -0.002 | 0.002 |
| MVPA T1 | TV T2 | 0.001 | 0.002 | 0.02 | 0.96 | -0.036 | 0.038 |
| MVPA T1 | PC/Gaming T2 | 0.001 | 0.03 | 0.004 | 0.48 | -0.001 | 0.003 |
| PC/Gaming T1 | PC/Gaming T2 | 0.42 | 0.24 | 0.07 | <0.001 | 0.281 | 0.563 |
| PC/Gaming T1 | TV T2 | 2.19 | 0.09 | 1.05 | 0.004 | 0.128 | 4.244 |
| PC/Gaming T1 | MVPA T2 | 0.26 | 0.11 | 0.10 | 0.01 | 0.061 | 0.449 |
| PC/Gaming T1 | OP T2 | -0.06 | -0.06 | 0.04 | 0.13 | -0.142 | 0.018 |
| TV T1 | TV T2 | 0.49 | 0.33 | 0.06 | <0.001 | 0.370 | 0.606 |
| TV T1 | PC/Gaming T2 | 0.01 | 0.13 | 0.004 | <0.001 | 0.005 | 0.021 |
| TV T1 | MVPA T2 | -0.01 | -0.07 | 0.01 | 0.11 | -0.021 | 0.003 |
| TV T1 | OP T2 | <0.001 | -0.01 | 0.002 | 0.90 | -0.004 | 0.004 |
| OP T1 | OP T2 | 0.37 | 0.25 | 0.06 | <0.001 | 0.248 | 0.492 |
| OP T1 | MVPA T2 | 0.06 | 0.02 | 0.15 | 0.68 | -0.231 | 0.353 |
| OP T1 | PC/Gaming T2 | -0.36 | -0.13 | 0.11 | <0.001 | -0.568 | -0.144 |
| OP T1 | TV T2 | -0.94 | -0.02 | 1.57 | 0.55 | -4.029 | 2.141 |
| MVPA T2 | MVPA T3 | 9.86 | 0.24 | 1.69 | <0.001 | 6.548 | 13.162 |
| MVPA T2 | OP T3 | 0.01 | 0.02 | 0.01 | 0.62 | -0.020 | 0.034 |
| MVPA T2 | TV T3 | -0.65 | -0.04 | 0.73 | 0.37 | -2.090 | 0.784 |
| MVPA T2 | PC/Gaming T3 | 0.17 | 0.01 | 1.10 | 0.88 | -1.995 | 2.333 |
| PC/Gaming T2 | PC/Gaming T3 | 3.27 | 0.09 | 1.64 | 0.05 | 0.056 | 6.492 |
| PC/Gaming T2 | TV T3 | -1.87 | -0.08 | 1.09 | 0.09 | -4.001 | 0.271 |
| PC/Gaming T2 | MVPA T3 | -3.16 | -0.06 | 2.51 | 0.21 | -8.083 | 1.757 |
| PC/Gaming T2 | OP T3 | -0.001 | -0.001 | 0.02 | 0.98 | -0.042 | 0.040 |
| TV T2 | TV T3 | 0.27 | 0.17 | 0.07 | <0.001 | 0.129 | 0.415 |
| TV T2 | PC/Gaming T3 | 0.60 | 0.25 | 0.11 | <0.001 | 0.386 | 0.818 |
| TV T2 | MVPA T3 | -0.15 | -0.04 | 0.17 | 0.39 | -0.475 | 0.183 |
| TV T2 | OP T3 | -0.001 | -0.05 | 0.00 | 0.33 | -0.003 | 0.001 |
| OP T2 | OP T3 | 0.14 | 0.19 | 0.03 | <0.001 | 0.077 | 0.211 |
| OP T2 | MVPA T2 | 0.17 | 0.002 | 4.12 | 0.97 | -7.892 | 8.238 |
| OP T2 | TV T3 | 0.78 | 0.02 | 1.79 | 0.66 | -2.723 | 4.279 |
| OP T2 | PC/Gaming T3 | 0.04 | 0.001 | 2.69 | 0.99 | -5.237 | 5.319 |

Please note that PC/Gaming T1, PC/Gaming T2, and MVPA T2 were square-root transformed. MVPA = moderate to vigorous physical activity [minutes per week]; OP = Outdoor play [days per week]; PC/Gaming = Computer use and gaming [minutes per day]; TV = TV and video watching [minutes per day]

*Squared multiple correlations (R^2^)*

| **Outcome** | **Estimate** |
| --- | --- |
| TV/Video watching T2 | 0.129 |
| PC/Gaming T2 | 0.118 |
| Outdoor play T2 | 0.071 |
| Moderate-to-vigorous physical activity T2 | 0.055 |
| TV/Video watching T3 | 0.027 |
| PC/Gaming T3 | 0.087 |
| Outdoor play T3 | 0.041 |
| Moderate-to-vigorous physical activity T3 | 0.066 |

MVPA = moderate to vigorous physical activity [minutes per week]; OP = Outdoor play [days per week]; PC/Gaming = Computer use and gaming [minutes per day]; TV = TV and video watching [minutes per day]
